# Symbolic Important Point Perceptually and Hidden Markov Model Based Hydraulic Pump Fault Diagnosis Method

**DOI:** 10.3390/s18124460

**Published:** 2018-12-17

**Authors:** Yunzhao Jia, Minqiang Xu, Rixin Wang

**Affiliations:** School of Astronautics, Harbin Institute of Technology, Harbin 150001, China; wangrx@hit.edu.cn

**Keywords:** Perceptually Important Point, Hidden Markov Model, hydraulic pump, fault diagnosis

## Abstract

Hydraulic pump is a driving device of the hydraulic system, always working under harsh operating conditions, its fault diagnosis work is necessary for the smooth running of a hydraulic system. However, it is difficult to collect sufficient status information in practical operating processes. In order to achieve fault diagnosis with poor information, a novel fault diagnosis method that is the based on Symbolic Perceptually Important Point (SPIP) and Hidden Markov Model (HMM) is proposed. Perceptually important point technology is firstly imported into rotating machine fault diagnosis; it is applied to compress the original time-series into PIP series, which can depict the overall movement shape of original time series. The PIP series is transformed into symbolic series that will serve as feature series for HMM, Genetic Algorithm is used to optimize the symbolic space partition scheme. The Hidden Markov Model is then employed for fault classification. An experiment involves four operating conditions is applied to validate the proposed method. The results show that the fault classification accuracy of the proposed method reaches 99.625% when each testing sample only containing 250 points and the signal duration is 0.025 s. The proposed method could achieve good performance under poor information conditions.

## 1. Introduction

Hydraulic pump plays an important role in the smooth running of hydraulic system, faults of hydraulic pump may cause a severe loss of life and property. Thus, the fault diagnosis work of hydraulic pump is necessary. It can help in improving reliability, reducing maintenance costs, and avoiding catastrophic accident. However, prior researches on hydraulic pump mainly focus on the design, manufacturing, and dynamic analysis. There is little research on fault diagnosis and maintenance. Under these circumstances, this paper focuses on fault diagnosis research of hydraulic pump and a data-driven fault diagnosis method for hydraulic pump based on Symbolic Perceptually Important Point (SPIP) and Hidden Markov Model (HMM) is proposed.

Hydraulic pump is typical rotating machinery, in recent years, data-driven fault diagnosis methods have been widely used in rotating machinery fault diagnosis. Various kinds of monitoring signals, such as vibration, pressure, voltage, and torque are used in status monitoring and fault diagnosis. Time-domain and frequency-domain features, such as variance, root mean square, kurtosis factor, impulse factor, energy, defective frequency, and its harmonics component are chosen as the main features because of their simple and definite physical meaning [1]. On one hand, these features are directly used as the status indicators of mechanical system, such as the increasing of kurtosis factor and root mean square may indicate anomalies in the system. On the other hand, these features are serving as input of classifiers, such as Artificial Neural Network (ANN), Support Vector Machine (SVM), Laplace Projection (LLP), Hidden Markov Model (HMM), and so on. These methods can achieve good performance without thoroughly mastering the physical model of diagnostic object [2]. However, most existing methods pay attention to statistical characteristics, spectrum analysis, decomposition, and reconstruction of signals. Movement shape of signals is seldom used in fault diagnosis work. In some practical engineering applications, it is difficult to obtain sufficient monitoring data, and spectral analysis and statistical analysis are difficult to carry out. Especially for a hydraulic system, sensors are difficult to set up and the poor information condition is a challenge for fault diagnosis. So, the movement shape analysis of monitoring signal should be paid more attention.

Time series symbolization is an effective way to do the movement shape research. Time series data, due to their numerical and continuous nature, is difficult to process, analyze, and mine. However, these tasks will become easier when the data can be transformed into meaningful symbols. In recent years, many scholars devoted to the study of symbolic sequence, symbolic sequence is generated through a symbolization process which is treated as a transformation of the original time-series from the phase space into a symbolic space, such that no significant information is lost. The symbolic sequence still contains key information of the system, but noise disturbance is smaller [3]. Amol Khatkhate et al. make use of symbolic time-series analysis for the early detection of small anomalies resulting from fatigue crack damage in ductile alloys, the D-Markov model is applied to construct anomaly measure, the results show that the evolution of fatigue crack can be detected in advance of component failure [4]. Li Yongbo et al. proposed the early health monitoring method of rolling bearing based on Symbolic Dynamic Filtering (SDF) and Intrinsic Characteristic-scale Decomposition (ICD). SDF is used to extract the fault features for depicting bearing performance degradation. Then, a fault alarm is triggered using cumulative sum. Finally, the extracted abnormal signal is decomposed by ICD, and the kurtosis method is used to select the principal product component that contains most fault information [5]. A fault diagnosis method that is based on modified multi-scale dynamic entropy and minimum redundancy maximum relevance is used to identify the regularity of time series. The method can assess the dynamical characteristics over a range of scales and it is able to recognize the different fault type of planetary gearboxes [6].

The results of symbolic sequence analysis are mainly determined by the symbolization algorithm and phase space trajectory of time series. It is noticeable that if all the points in original time series are used in analysis, without filtering before the symbolization process, the symbolic series may be over long, causing a negative impact on computational efficiency and noise disturbance. Therefore, the original time series must be compressed with as little information loss as possible. A lot of works have been carried out in time series compression. Christos Yiakopoulis et al. utilize Piecewise Aggregate Approximation (PAA) for the analysis of signals, the original signal is divided into a number of equal subsequences, then each subsequence is substituted with its mean value, and a low dimension series is achieved [7]. G. Das et al. propose a simple N/S conversion, a fixed length window is used to segment a time series into subsequences and the time series is then represented by the primitive shape patterns that are formed [8]. T. Liu combines the zero-crossing feature (ZC) with the Hidden Markov Model for bearing performance assessment, the ZC feature is a time domain feature that can be extracted from time series at a low cost and it carries the frequency information as well [9]. However, these methods may cause severe information loss, which may have an adverse impact on subsequent analysis.

In order to solve the aforementioned problems, this paper proposes a hydraulic pump fault diagnosis method that is based on the Symbolic Perceptually Important Point and Hidden Markov Model. An adaptive method, named Perceptually Important Point, is applied to compress original time series. The method looks for data points that have greater influence on the overall movement shape of original time series, these points are called important point. Subsequently, original series is transformed into a low dimension PIP series that is constructed by a number of important points in the original series [10]. PIP series can depict the movement shape of original series clearly and greatly reduce the information loss in the compression process of time series. Afterwards, PIP series is transformed into symbolic series, the Genetic Algorithm is imported to optimize the partition scheme in order to take advantage of distribution information of original series, maximizing the difference between reference symbolic series from different operating conditions. After that, HMM is applied to recognize different operating conditions and symbolic series of reference signal serves as training data. Both the symbol of each important point and transformation rules between adjacent important points can be utilized by HMM, this characteristic can help in significantly reducing the information requirement. When a testing sample is inputted in models, the likelihood value outputted would serve as the metric of fault classification. At last, a fault simulation experiment is used to validate the performance of the proposed method and vibration signal is used as the fault indicator of hydraulic pump. The results show that the classification accuracy reaches 99.625% when each testing sample contained 250 points and the signal duration is 0.025 s. The information requirement of the proposed method is far less than the existing methods.

The innovation of this paper can be summarized, as follows:

- Perceptually Important Point technology is firstly imported into the fault diagnosis field. The method can compress monitoring signals with little information loss, depicting the overall movement shape of original signal accurately.
- Genetic Algorithm is applied to optimize the symbolic space partition scheme; the distribution of important points can be used to enlarge the difference between symbolic series of different operating conditions [11].
- Perceptually Important Point technology is combined with the Hidden Markov Model, good performance can be attained with a significantly less information requirement than existing methods.

The remaining part of this paper is organized, as follows: Perceptually Important Point technology, time series symbolization process, and Hidden Markov Model are introduced in Section 2. In Section 3, the scheme of hydraulic pump fault diagnosis method that is based on Symbolic Perceptually Important Point technology and the Hidden Markov Model is shown. In Section 4, a case study is presented to validate the performance of proposed method and the results are given. Finally, the conclusion is given in Section 5.

## 2. Methods

Monitoring signals of the mechanical system are mainly determined by the physical model of the system, and it may be affected by equipment degradation, operating condition, and external environment factors. Once the status of equipment changes, for example, normal deterioration or mechanical failure, the signal would change as well. Monitoring signals are always digital signals that can be regarded as time series, in this section, the compressing, symbolization, and classification procedure of time series are introduced.

### 2.1. Perceptually Important Point

Time series is constructed by a sequence of data points and the value of each point has different degrees of influence on the movement shape of time series. That is, each data point has its own importance to the time series, a data point may determine the overall movement shape of the time series, while another only has little influence on the time series or it can even be discarded. Perceptually Important Point technology attempts to look for the point that has key influence on the overall movement shape of time series [12,13,14,15].

**Figure 1:**
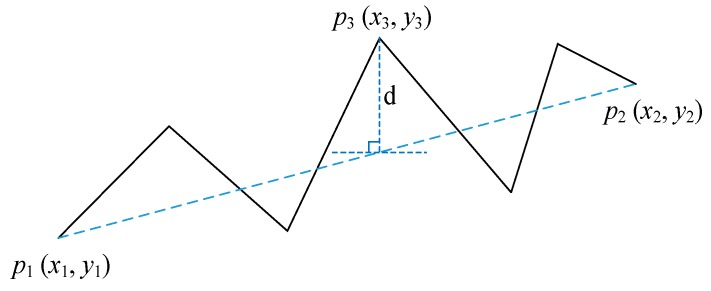
The vertical distance from the point to the line connecting adjacent important points.

According to the PIP framework, a time series *X* = [*x*_1_, *x*_2_, *x*_3_, …, *x_n_*] can be represented by a PIP series *P* = [*p*_1_, *p*_2_, *p*_3_, …, *p_m_*], *m* << *n*. The first two important points, *p*_1_ and *p*_2_ are the first and the last point of original series, *x*_1_ and *x_n_*, the next important point *P*_3_ is the point with the maximum impact on the movement shape of original time series among the remaining points in original time series. The influence is measured by the vertical distance from the point to the line connecting its adjacent important points; that is to say, the third important point is the point with the maximum vertical distance to the line connecting *x*_1_ and *x_n_*. The forth important point is that the point remains in *X* with maximum distance to its adjacent important points, *p*_1_ and *p*_2_ or *p*_2_ and *p*_3_. The process of locating the important points continues until getting *m* important points, the points that were identified in the earlier iterations are considered to be more important than points identified later [16]. The process of measuring the distance to adjacent important points is depicted in Figure 1, the curve is a time series includes six points *X* = [*x*_1_, *x*_2_, *x*_3_, *x*_4_, *x*_5_, *x*_6_], the first and the last point are regarded as *p*_1_ and *p*_2_. Firstly, the slope of the line connecting *p*_1_ and *p*_2_ is calculated by Equation (1), then the vertical distance between the remaining points to the line connecting their adjacent important points *d_i_* is calculated by Equation (2), the point with maximum vertical distance is regarded as *p*_3_. During the process of determining the forth important point, the vertical distance of points between *p*_1_ and *p*_3_ is the vertical distance from the point to the line connecting *p*_1_ and *p*_3_, the vertical distance of the points between *p*_2_ and *p*_3_ is the distance from the point to the line connecting *p*_2_ and *p*_3_. After all of the important points are determined, the order of important points will be rearranged according to their index in original time series, the series obtained is PIP series. The PIP series can depict movement shape of original time series and it will replace the original time series in subsequent analysis. Table 1 shows the scheme of Perceptually Important Point technology.

(1) $$k=(y_{2}-y_{1})/(x_{2}-x_{1})$$

(2) $$d_{i}=\left|y_{1}+k\times (x_{i}-x_{1})-y_{i}\right|$$

where *x_i_*, *y_i_* is the horizontal ordinate and vertical ordinate of the points in the original series, *d_i_* is the vertical distance of *x_i_*.

**Figure 2:**
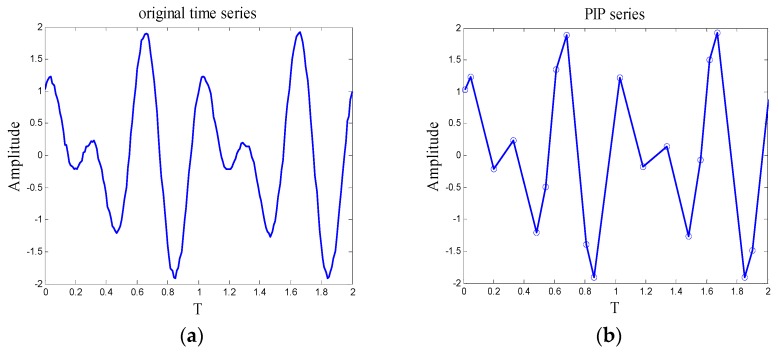
(**a**) Waveform of simulating signal; (**b**) Waveform of PIP series.

Here, a simulating signal that is generated by MATLAB is imported to validate the performance of the algorithm, Figure 2a is the waveform of simulating signal. The expression is *x* = sin(4 × pi × *t*) + cos(6 × pi × *t*), and 10% Gaussian white noise is added, the series includes 200 points, the time interval between each point is 0.01 s. Subsequently, the time series is compressed by PIP technology, Figure 2b is the waveform of PIP series, the length of PIP series is 20. As can be seen from the results of time series compression, when the density of important points is one-tenth of the original time series, the overall movement shape of the simulating signal can be depicted by PIP series clearly.

The time series compression process must cause information loss that always has a negative impact on time series analysis. In order to clarify the applicability of time series compression methods and the influence of the length of PIP series, this paper proposes a metric of information loss, called reconstruction error. For each point in original series, there is a corresponding point on the PIP curve (may not be important point), the horizontal ordinate of these two points is equal and the vertical ordinate of the corresponding point *x_i_* can be attained by Equation (3).

(3) $$c_{i}=y_{L}+(x_{i}-x_{L})(y_{R}-y_{L})/(x_{R}-x_{L})$$

where *c_i_* is vertical ordinate of the corresponding point of *x_i_*, *x_L_*, *x_R_*, *y_L_*, *y_R_* is vertical and horizontal ordinate of the left and the right important point of *x_i_*, the corresponding point of *x_i_* can be expressed as (*x_i_*, *c_i_*). The ratio of deviation *e_i_* can be calculated by Equation (4), it is defined as the local reconstruction error, the mean value of local reconstruction error in a time series is defined as reconstruction error, it can be attained by Equation (5). The reconstruction error reflects the residual level between the PIP series and the original series.

(4) $$e_{i}=\left|c_{i}-y_{i}\right|/\left|y_{i}\right|$$

(5) $$E=(1/m)\sum_{i=1}^{m}e_{i}$$

**Figure 3:**
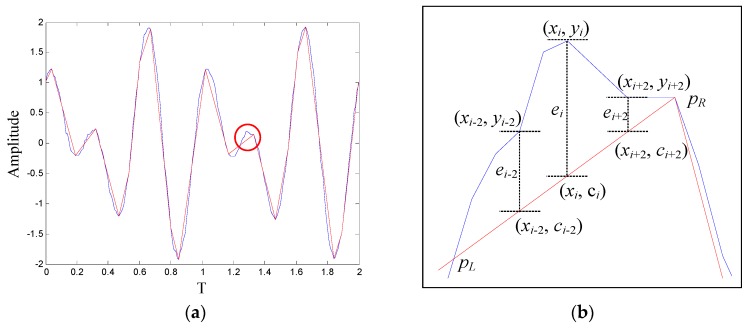
(**a**) Schematic diagram of reconstruction error calculation; (**b**) Local enlarge of the marked region in (**a**).

**Figure 4:**
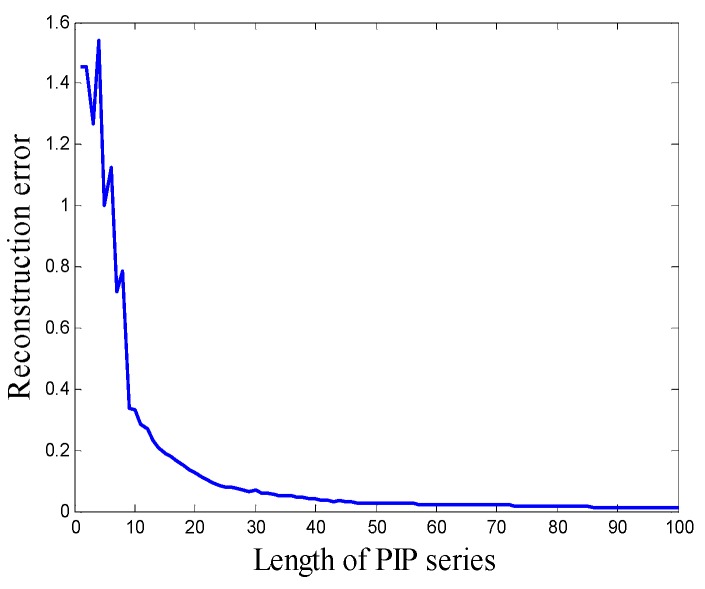
The relation between reconstruction error and the length of PIP series.

The procedure of calculating the reconstruction error is depicted in Figure 3a, the blue line is the original series, and the red line is the waveform of PIP series. Figure 3b is the local enlarged view of the region marked in Figure 3a, $e_{i}$ is the local reconstruction error of point *x_i_*, after the local reconstruction error of all the points are calculated, the reconstruction error *E* can be attained by Equation (5). In Figure 4, the relation between the reconstruction error and the length of PIP series is given. It can be seen that the reconstruction error of simulating signal is extremely high when the length of PIP series is short. With the increasing of the length, the reconstruction error recesses sharply, after the length exceeds 25, the recession tendency slows down, over length PIP series may aggravate noise disturbance and reduce the computational efficiency. Therefore, in this case, compressing the original signal into a PIP series including 20–30 points is appropriate. How to determine the density of important points in the practical application process will be discussed in Section 4.

### 2.2. Time Series Symbolization

Time series symbolization is treated as a transformation of original time series from the phase space into a symbolic space. It is performed by partitioning the time series into a finite set of segments that are labeled as symbols. The procedure can help in reducing the disturbance of environmental factors, facilitating pattern recognition, and increasing computational efficiency. The scheme of PIP series symbolization is introduced this section.

First, the symbolic space is partitioned, the mean value *μ* and standard deviation *σ* of PIP series should be calculated, and the number of regions in symbolic space *k* should be determined. Mean value *μ* serves as the center of symbolic space, fractiles of standard deviation serve as region boundaries. The space is partitioned into *k* regions with a set of fractiles of standard deviation *F* = [*f*_1_, *f*_2_, …, *f_k_*], *f_i_*> *f_i_*_−1_ > 0. Each region is labeled with a symbol, the number of symbol is equal to the amount of region in symbolic space *k*, and the symbol set can be expressed as *SY* = [*sy*_1_, *sy*_2_, …, *sy_k_*]. Second, important points are transformed into symbols according to their location in symbolic space. Each important point is encoded with the symbol corresponding to the region it locates in, then the PIP series *P* = [*p*_1_, *p*_2_, *p*_3_, …, *p_m_*] is transformed into symbolic series *S* = [*s*_1_, *s*_2_, *s*_3_, …, *s_m_*].

**Figure 5:**
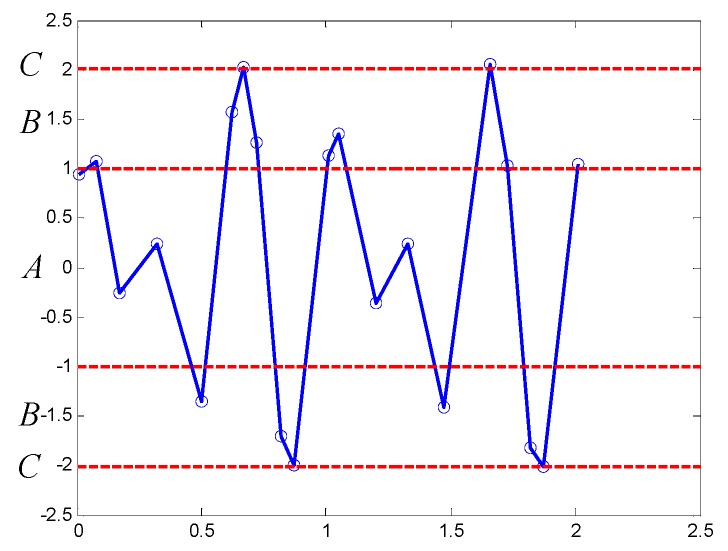
Symbolization results of simulating signal based on 3σ criterion.

Here, an example is applied to explain the procedure, simulating signal in 2.1 is transformed into symbolic series based on 3σ criterion, the PIP series of the simulating signal is expressed as *P* = [*p*_1_, *p*_2_, …, *p*_20_]. The mean value *μ* = 0.0696, the standard deviation *σ* = 1.0323, and the number of regions *k* = 3, fractiles of deviation set *F* = [1, 2, 3], and the symbol set *SY* = [A, B, C]. The results are shown in Figure 5, red dotted lines are partition boundaries, three symmetric regions are labeled with A, B, and C. All of the points in *P* are encoded with the symbol corresponding to the region that they are located in. For instance, the value of the first and the second points is 1.004 and 1.213, so they are encoded with A and B, the whole symbolic series obtained is ABAABBCBBBBBAABCBBBB.

In practical fault diagnosis work, the symbolization scheme has key influence on the classification accuracy. Symbolic series from different operating conditions may show great difference that is based on a symbolization scheme and may be almost identical based on another scheme. There are two major symbolization rules: uniform partitioning and maximum entropy rule, but none of them take advantage of distribution information of the original series. In practical application, determining the partition scheme with the distribution characteristics of PIP series will help in maximizing the difference between symbolic series of different operating conditions, thus improving classification accuracy. This paper imports Genetic Algorithm in searching the optimal symbolic space partition scheme, maximizing the difference between reference series from different operating conditions, and reducing the probability of confusion.

**Figure 6:**
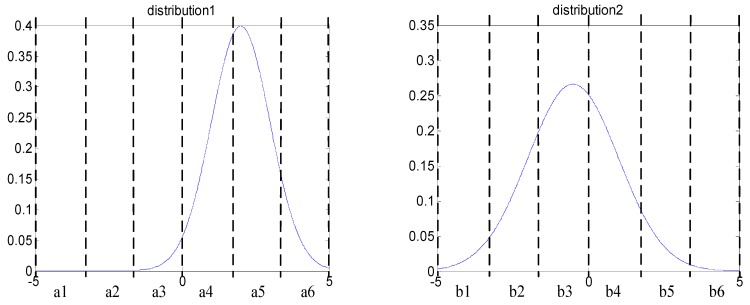
Probability density distribution of important points.

Two simulating signals are used to elaborate the procedure of searching optimal scheme. First, they are compressed into PIP series, the probability density distribution curve of important points are shown in Figure 6. It can be seen that the distribution of important points is quite different, so the difference must be keep in the symbolization procedure. The Genetic Algorithm has the ability to achieve this goal.

The symbolic space is divided into six region, the amount of important points in each region are expressed by *a*_1_–*a*_6_ and *b*_1_–*b*_6_, and two distribution vectors can be obtained: *A* = [*a*_1_, *a*_2_, …, *a_i_*], *B* = [*b*_1_, *b*_2_, …, *b_i_*], *i* = 6. Then, the Genetic Algorithm is used to determine partition nodes, and the reciprocal of Euclidean distance between vectors is chosen as the fitness function of Genetic Algorithm; the fitness function is shown in Equation (6). A smaller fitness function value indicates that there is bigger difference between the two reference symbolic series. The difference between two reference symbolic series is regarded as the largest according to the partition scheme that was obtained by the Genetic Algorithm.

It is noticeable that if the amount of each symbol is unbalance, data underflow phenomenon may appear and the computational efficiency may be reduced. Thus, the liner constraint must be added based on the characteristic of distribution, the liner constraint of the example is that the width of each region is no less than 0.5. The algorithm of time series symbolization is shown in Table 2.

(6) $$Fit=1/\sqrt{\sum_{i=1}^{n}a_{i}^{2}-b_{i}^{2}}$$

where *Fit* is the value of fitness function.

### 2.3. Hidden Markov Model

Hidden Markov Model (HMM) is an effective tool to characterize a time series, which is initially introduced and studied in the late 1960s [17]. It has a wide range of applications in the field of speech recognition, economic analysis, and mechanical engineering due to its strong mathematical basic theory and well developed algorithms [18,19]. The components of HMM can be described, as follows:

1. Hidden states: the hidden states are defined as *W* = [*w*_1_, *w*_2_, ..., *w_M_*], and the state in the time *t* is defined as *q_t_*;
2. Observations: the observations is the real output of system. Let *V* = [*v*_1_, *v*_2_, …, *v_N_*] be the set of observation symbols, and the observation at time *t* is defined as *o_t_*;
3. State transition probability matrix *H*: $h_{ij}=P(q_{t+1}=w_{j}|q_{t}=w_{i})$, 1≤i, j≤M, the sum of each row is 1;
4. The observation probability vector *L*: $l_{j}(k)=P(o_{t}=v_{k}|q_{t}=w_{j})$, 1≤j≤M, 1≤k≤N, the sum of the vector is 1; and,
5. The initial state distribution π: $\pi_{i}=P(q_{1}=w_{i})$, 1≤i≤M.

**Figure 7:**
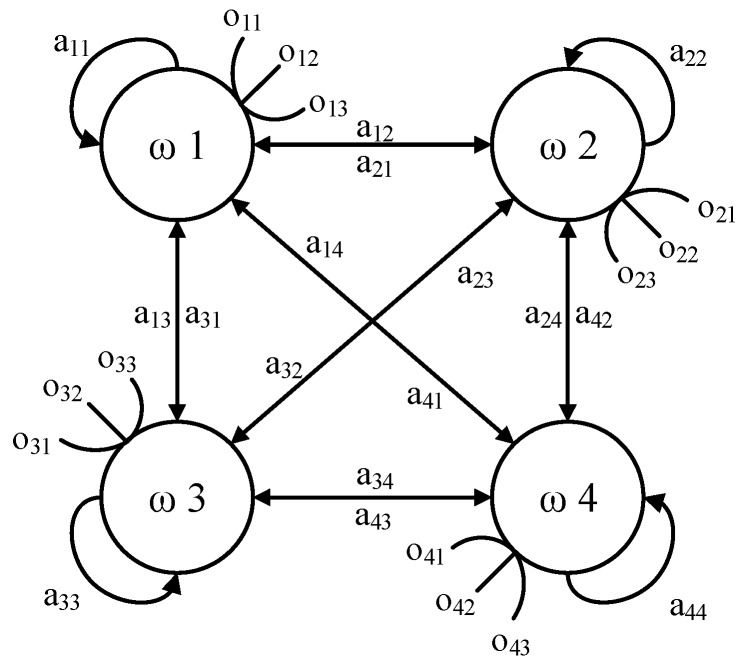
Topological structure of the Hidden Markov Model.

Subsequently, the HMM can be specified by π, *H,* and *L*, the model can be expressed as λ=(π,H,L), the topological structure of HMM is elaborated in Figure 7. Three assumptions are associated with the using of the HMM theory [20]:

1. The probability of the state at a given time *t* only depends on the state of previous time *t* − 1;
2. The state transition probabilities are independent of the actual time at which the transition occurs; and,
3. The current observation only depends on the current state and it is independent of the previous observations.

There are three basic algorithms in HMM, Forward–Backward procedure, Viterbi algorithm, and Baum-Welch algorithm. The Forward–Backward procedure is applied to estimate the probability of the observed sequence is generated by a given model λ; Viterbi algorithm is applied to estimate the optimal state sequence *Q* = (*q*_1_, *q*_2_, …, *q_t_*) when a model λ and a observation sequence *O* = (*o*_1_, *o*_2_, …, *o_t_*) are given; the Baum–Welch algorithm is used for HMM parameters re-estimation, outputting parameters λ=(π, H, L) to maximize the probability of the given observation sequence [21]. The advantage of HMM is that both the symbol of each important point and transformation rules between adjacent points can be utilized in the process of series analysis; more information can be mined from original time series. Therefore, HMM needs less monitoring information than other methods in fault classification.

In the process of hydraulic pump fault diagnosis, models correspond with different operating conditions is first established with reference symbolic series using the Baum–Welch algorithm. Subsequently, testing samples are inputted in models, the operating condition corresponds with the model outputting the maximal probability is regarded as the operating condition of testing samples.

## 3. Fault Diagnosis Framework

The process of fault diagnosis method based on Symbolic Perceptually Important Point and the Hidden Markov Model is described, as follows:

1. Collecting monitoring signals of equipment in different operating conditions, establishing a reference data set;
2. Extracting PIP series using Perceptually Important Point technology;
3. Transforming PIP series into symbolic series, Genetic Algorithm is applied to optimize the partition scheme of symbolic space;
4. Reference symbolic series of different operating conditions is used to train HMM and models corresponding with each operating conditions can be attained; and,
5. Unlabeled testing samples are transformed into symbolic series and inputted into the models obtained above. When comparing the outputting likelihood of each model, the operating condition corresponding with the model outputting maximal likelihood is regarded as the operating condition of testing samples.

**Figure 8:**
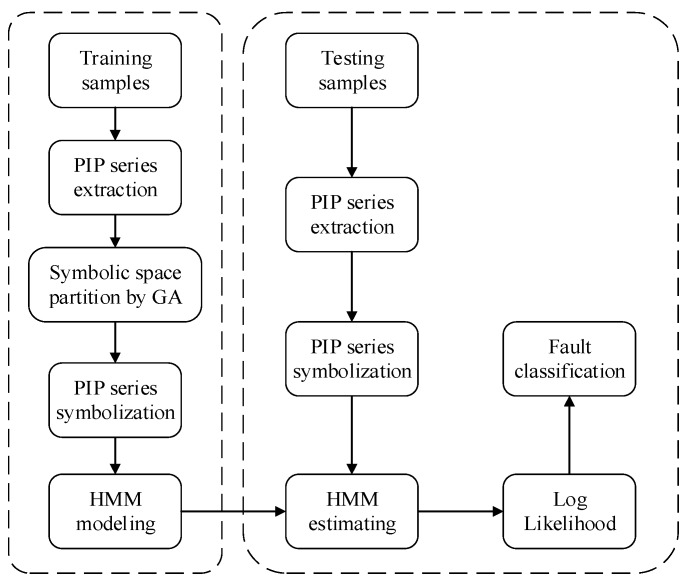
Hydraulic pump fault diagnosis framework.

The scheme of the proposed method is shown in Figure 8.

## 4. Case Study and Results

### 4.1. Experiment Setup

**Figure 9:**
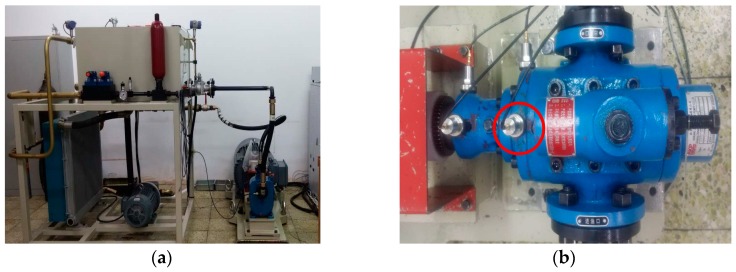

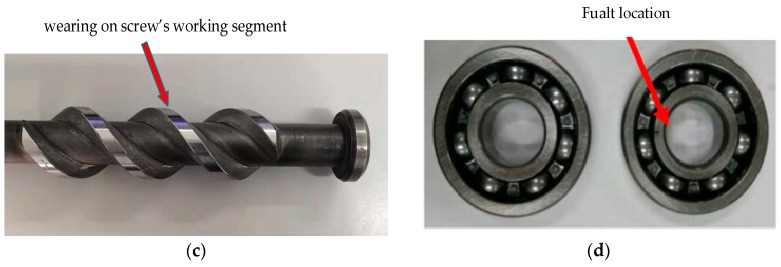
(**a**) Experiment rig; (**b**) Sensor location; (**c**) Fault screw; and, (**d**) Fault rolling bearing.

In order to demonstrate the validity of the proposed method, a fault simulation experiment is conducted in the Harbin Institute of Technology. The test rig is shown in Figure 9a, the type of testing pump is AKP-032/084 three-screw pump, and a deep grove ball bearing 6205 is used for supporting the screw. The hydraulic system is driven by a 55 kW three-phase asynchronous motor with a nominal speed of 3250 rpm, and a frequency converter is used to control the motor’s speed. Hydraulic medium in the hydraulic system is L-HM-32 hydraulic oil, the kinematic viscosity is 80 m^2^/s when the temperature is 20 °C. In order to simulate the influence of load, the pressure at the oil return tube is set to 1 Mpa. The vibration signal is collected by a velocimeter with a bandwidth of 4–4000 Hz and sensitivity of $4\;\;\mathrm{mV}/\mathrm{mm}\cdot s^{-1}$, being located on the front cover of hydraulic pump; the location of sensor is shown in Figure 9b.

Crack of rolling bearing and wearing on screw’s working segment are common faults of the three-screw pump. Rolling bearing crack is mainly caused by the misalignment of coupling and lack of lubrication. Wearing on screw’s working segment often appears when hydraulic oil is polluted by grain impurity or when the operating temperature is too high. The kinematic viscosity will decrease with the increasing of operating temperature, reducing the lubricating capacity of hydraulic oil. Crack of rolling bearing may cause abnormal vibration and noise, wearing of screw will lead to a decrease in mechanical efficiency. In order to simulate practical diagnosis work, four operating conditions: nominal condition, 0.2 mm wearing on screw’s working segment, 0.4 mm wearing on screw’s working segment, and rolling bearing inner race crack are imported to evaluate the classification accuracy of the proposed method. The processing method for the test specimen of screw wearing is grinding and for rolling bearing inner race crack is wire–electrode cutting. The diagram of fault screw and rolling bearing are shown in Figure 9c,d.

A data acquisition system NI PXI-8880 is used to collect the vibration signal, the sampling rating is 10,000 Hz and the speed of rotation motion is set to 2900 rpm. In this experiment, the hardware environment is INTEL i3 3.6 GHz, 8 GB RAM, and the software environment is WINDOWS7 64 bit operating system, MATLAB 2014a.

**Figure 10:**
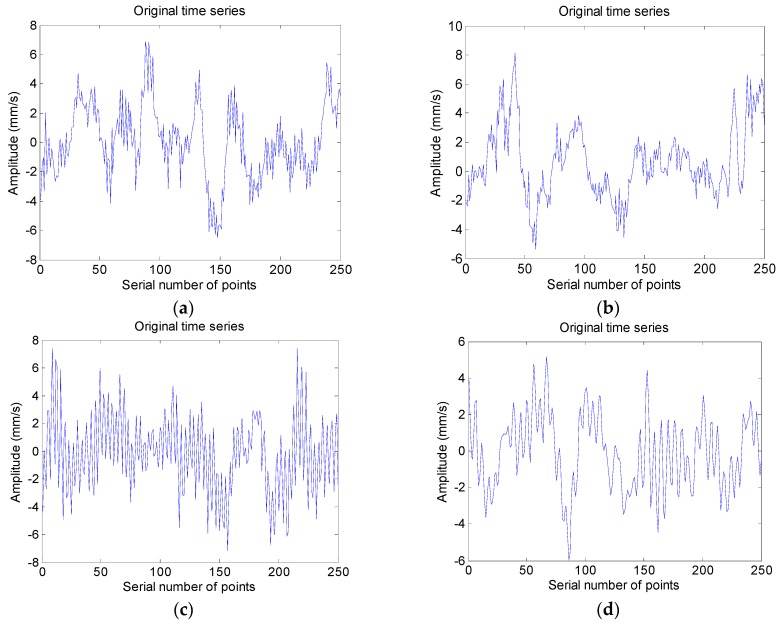
Time domain waveform; (**a**) Condition 1; (**b**) Condition 2; (**c**) Condition 3; and, (**d**) Condition 4.

**Figure 11:**
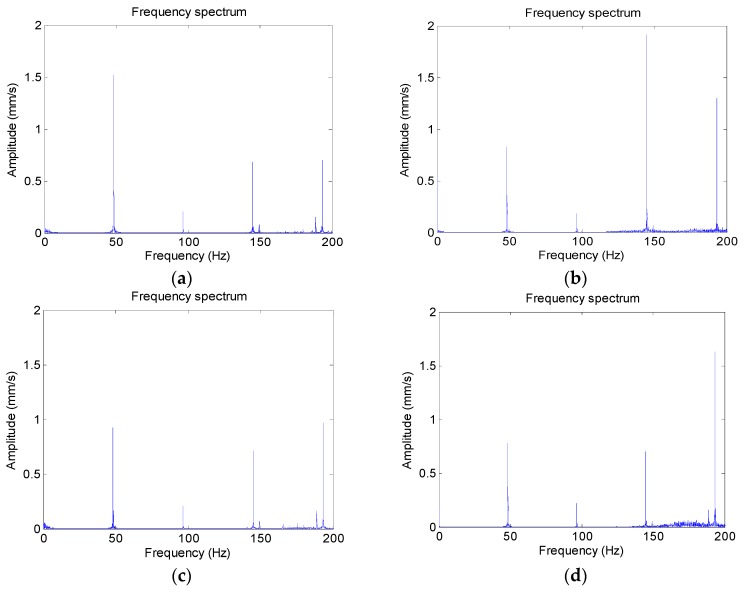
Frequency spectrum of each operating condition; (**a**) Condition 1; (**b**) Conditon 2; (**c**) Condition 3; and, (**d**) Condition 4.

The vibration signal of each operating condition is collected, the duration of signals is 15 s, and the length of signals is 150 k data points. Initially, the vibration-based Fast Fourier Transformation is used to extract fault frequency. Figure 10 and Figure 11 are the time domain waveform and the frequency spectrum of each operating condition, the frequency resolution is 0.067 Hz, and the physical quantity of vertical ordinate of time domain waveform and frequency spectrum is $\mathrm{mm}\cdot s^{-1}$. Fault frequency for screw wearing is 48.33 Hz and for bearing inner race crack it is 213.92 Hz. It can be seen that the frequency spectrum of each condition is resembled, and identifying different operating conditions by the frequency spectrum is so difficult, especially for faults on screw. Accordingly, the SPIP and HMM based fault diagnosis method is used for fault recognition. The signal of each operating condition is divided into 600 samples, each sample includes 250 points, and the signal duration is 0.025 s; one-third of them are used to train the HMM and the rest are used for testing. Detailed information of the operating condition is shown in Table 3.

### 4.2. Creation of Symbolic Series

**Figure 12:**
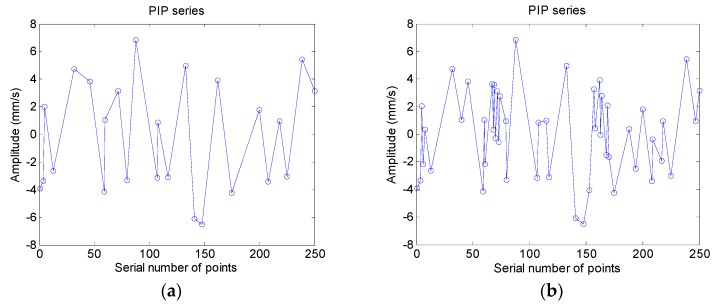
Waveform of PIP series (**a**) PIP series with 25 points; (**b**) PIP series with 50 points.

First, the original time series of all of the samples are transformed into PIP series through the use of Perceptually Important Points technology. Figure 12a is PIP series of a sample from nominal condition, the length of PIP series is set to 25 and in Figure 12b is 50. It can be seen that the PIP series can depict the movement shape of original data series clearly when the length is 25, after the length increase to 50, the PIP series is affected by the noise and signal modulation significantly. In this case, the length of PIP series is set to 25 temporarily; the influence of the length of PIP series on classification accuracy and computational efficiency will be discussed later.

**Figure 13:**
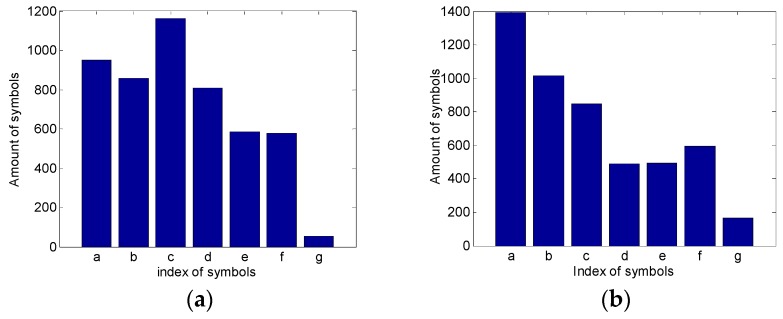

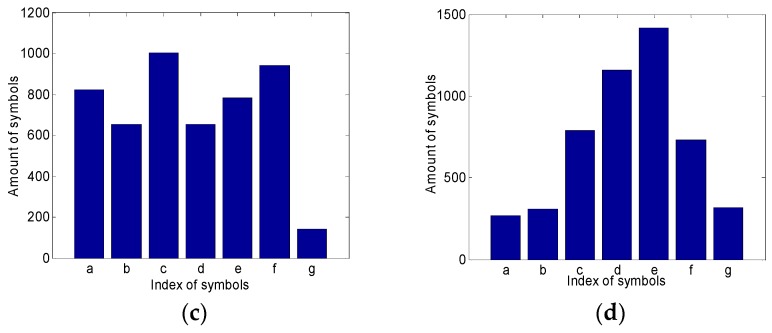
Symbol distribution of each operating condition: (**a**) Condition 1; (**b**) Condition 2; (**c**) Condition 3; and, (**d**) Condition 4.

Subsequently, the PIP series are transformed into symbolic series according to the location of important points in the symbolic space, the symbolic space is divided into seven parts and each part is labeled with a symbol a–g. The Genetic Algorithm is used to optimize the partition of symbolic space, the results show that when the partition nodes are 0.68, 1.04, 1.41, 1.75, 2.2, 3 (fractiles of standard deviation), the smallest fitness function can be attained. Figure 13 is total amount of each symbol in the reference data set of each operating condition. It shows that after being optimized by Genetic Algorithm, the distribution of symbols changes significantly when the status of equipment changes.

### 4.3. Fault Classification

After the symbolic series of all the samples are obtained, the Hidden Markov Model is trained with 200 samples corresponding to each operating condition, state transition probability matrix, observation probability vector, and initial state distribution can be got.

Afterwards, models are used to recognize operating conditions and 400 testing samples of each condition are inputted into each model, the log likelihood outputted is the metric of the input series is generated by the given model. For a given symbolic series *S* = [*s*_1_, *s*_2_, *s*_3_, …, *s_m_*] and a given model λ=(π,H,L), the value of likelihood is the probability that *S* is generated by the model λ=(π,H,L). It can be expressed as Equation (7) and log likelihood can be obtained by Equation (8). Higher output value indicates a higher matching degree between the testing samples and reference series. Thus, when a testing sample is inputted into all the models, the operating condition corresponding to the model that outputs the maximum probability value is regarded as the operating condition of the testing sample.

(7) $$Likelihood=P(o_{1}=s_{1},o_{2}=s_{2},\ldots ,o_{m}=s_{m}|\lambda )$$

where *o_i_* 1≤i≤m, are the observations of the Hidden Markov Model.

(8) $$Log_{}Likelihood=\mathrm{lg}(Likelihood)$$

**Figure 14:**
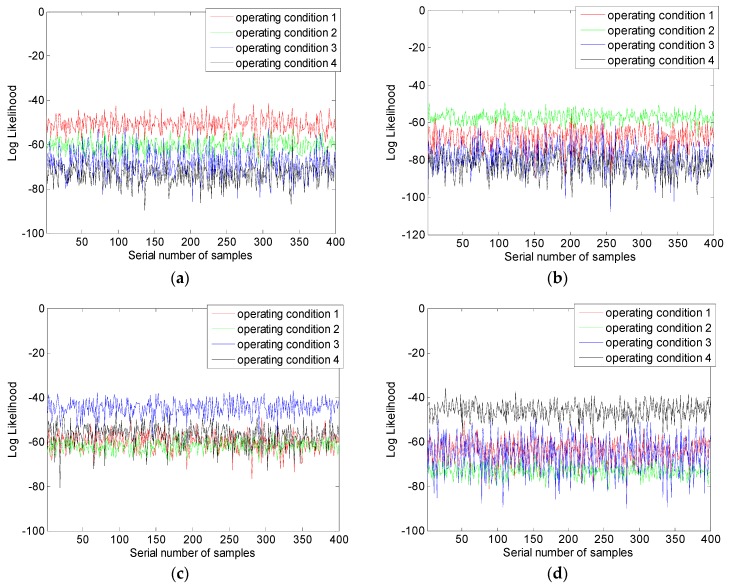
Classification results of the method: (**a**) Model 1; (**b**) Model 2; (**c**) Model 3; and, (**d**) Model 4.

Figure 14 is the classification results of hydraulic pump fault data set, the x coordinate is a serial number of testing samples and the y coordinate is log likelihood outputted by models, and models corresponding to conditions 1–4 are defined as models 1–4. In Figure 14a, the likelihood is obtained by inputting testing samples of condition 1 into models corresponding to each operating condition. It can be seen that the value of log likelihood that is outputted by model 1 is significantly higher than the value outputted by other models, and the likelihood outputted by other models are confused seriously. Accordingly, most of testing samples of condition 1 can be classified correctly. Subsequently, input testing samples of other three conditions into all the models, the results are listed in Figure 14b–d. The results follow the rule given above as well. Average log likelihood that is outputted by models is listed in Table 4; the data show that when testing samples of condition 1 are inputted in model 1, the average log likelihood is −50.91. The value is much bigger than the likelihood outputted when the samples are inputted in the other three models, the value of them are −60.35, −68.21, and −73.34, and the data of other conditions has the same character. Accordingly, when the samples inputted in the model correspond to the true condition, the log likelihood outputted is larger than the likelihood that is outputted by other models. It is the core basis of fault classification.

Table 5 is the confusion matrix of the fault classification method, from the classification accuracy of the method reaching 99.625% can be found. There are little confusion phenomenon appearing between operating Conditions 1 and 2, operating Conditions 3 and 4. The reasons for this phenomenon include the disturbance of noise, over-fitting of models, and in the process of classification, the data length of each sample is so short, in this interval, the signal may exhibit the characteristics of similar condition.

The results of the experiment show that the SPIP and HMM based hydraulic pump fault classification method have good performance on the fault classification. The method can reach high classification accuracy based on a short length of vibration signal from a single channel, achieving fault recognition without spectrum analysis and statistical characteristic.

The performance is influenced by lots of factors, the main factors and their influence would be discussed later, and parameter optimization process would be explored.

### 4.4. Parameter Optimization

The main metric of classifiers’ property is classification accuracy and computational efficiency. In the proposed method, the number of symbol and the length of PIP series may affect the performance; the process of parameter optimization is discussed in this part.

In the practical application of parameter optimization, the length of the PIP series must be assumed before the optimization process. Afterwards, Bayesian Information Criterion (BIC) is used to determine the roughly range of the number of symbol. Classification accuracy and computational efficiency of the method are calculated when the number of symbols is in this scope and the number of symbols is determined after weighing classification accuracy and computational efficiency. Once the number of symbol is decided, the length of PIP series should be adjusted. The performance of the method with different length of PIP series should be revalued and the length with the best performance is chosen as the new length of PIP series. The parameter optimization process of fault simulation experiment data set is shown later.

First, the influence of the number of symbol is considered and the length of PIP series is temporarily set to 25 and 50. Bayesian Information Criterion is imported to roughly determine the range. The Bayesian information criterion is mainly used for model choice, many parameter estimation problems select the likelihood function as the objective function. Increasing the number of model parameters will increase the likelihood function, improving the fitting accuracy, but an over complex model may cause over-fitting phenomenon. Thus, Bayesian information criterion introduces a penalty item that relates to the number of model parameters. The purpose is to make the model as simple as possible on the premise that without serious loss of classification accuracy, reducing the risk of over-fitting [22]. The expression of Bayesian information criterion is shown as Equation (9).

(9) B=2klnn−2lnl

where *k* is the number of model parameters, *n* is the number of samples, *l* is likelihood value, and *B* is the BIC value. The first item in Equation (9) reflects the complexity of the model, the second item is fitting precision of the model, and a smaller value of *B* shows better property of the model. Subsequently, the value of the proposed model is calculated, the results show that in this case, there is no significant gap in the value of *B* when the number of symbol between 4–9. Therefore, the classification accuracy and computational efficiency of the model are calculated when the number of symbol in the range from 4 to 9, and the results are shown in Table 6.

The results show that when the length of PIP series is 25, partitioning the symbolic space into seven parts, the model reaches the highest classification accuracy of 99.625%. When the length of series is 50, the partitioning symbolic space into five parts, the model reaches the highest classification accuracy of 99.875%. Continuing to increase the number of symbol could not significantly affect the accuracy, on the contrary, it may reduce the computational efficiency, weighing computational efficiency, and classification accuracy, and the number of symbol is set to 7.

**Figure 15:**
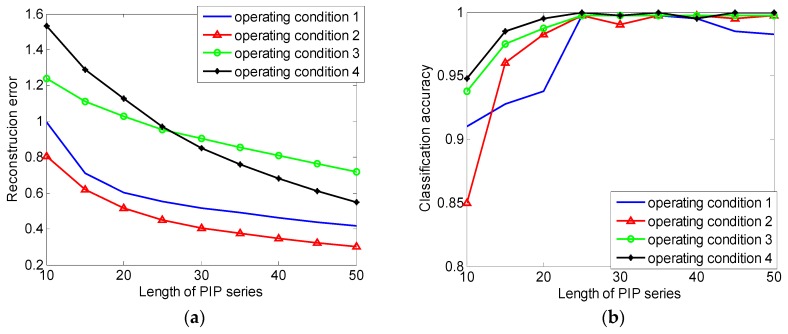
(**a**) The relation between classification accuracy and the length of PIP series; (**b**) The relation between reconstruction error and the length of PIP series.

Second, the influence of the length of PIP series is considered, the length over short will cause severe information loss and the length over long will cause over fitting phenomenon and reduce the computational efficiency. A proper length needs to be determined after weighing the classification accuracy, over-fitting phenomenon, and computational efficiency. Here, the construction error, classification accuracy, and computational efficiency are calculated when the length is 10 to 50, the results are shown in Table 7. The relation between reconstruction error, classification accuracy, and the length of PIP series are shown in Figure 15. It can be seen that when the length of PIP series is over 20, in other words, over 8% of the length of original data, the decline tendency of the curve decreases significantly. Lengthening the PIP series cannot effectively reduce information loss. When the length of PIP series less than 25, the classification accuracy increases with the increasing of the length rapidly and reaches 99.625% at 25. When the length of PIP series is longer than 25, the classification accuracy stops increasing. Time consumption data reflects that time consumption is proportional to the length of series and there is no sense to lengthen the PIP series when the length over 25, the length of PIP series is set to 25.

### 4.5. Performance Comparison

This section, the performance of the proposed method is compared with existing time series compression methods SAX (Symbolic Aggregate Approximation) and ZC (zero crossing characteristic features). They are two typical feature extraction methods that are based on movement shape analysis [23]. Samples in this part are same with aforementioned, each sample are divided into 25 subintervals, the SAX and ZC features are used to compress the original signal, and construct the feature series. HMM is used to recognize the operating conditions, 200 samples are used to train the model, and 300 samples are used for testing. Table 8 is the performance of these methods; the results show that SPIP and HMM based method has higher accuracy than similar methods.

Subsequently, the performance of the proposed method is compared with the commonly used machine learning methods that are based on statistical learning, including BP Neural Network (BPNN), Radial Basis Function Neural Network (RBFNN), and Support Vector Machine (SVM) [24,25,26,27]. In this process, original signal is divided into a number of subintervals with the length of 5000 points, a total of 100 training samples, and 180 testing samples are obtained for each operating condition. Feature vector is formed by mean, variance, effective value, kurtosis value, energy, skewness, defective frequency, and its harmonics component of the original signal. Table 9 is the classification accuracy and computational efficiency of the machine learning methods. The results show that the proposed method is superior to BPNN and RBFNN, slightly inferior to SVM in classification accuracy, but its information requirement is far less than statistical learning methods and the scope of application is broader.

## 5. Conclusions

In order to achieve hydraulic pump fault diagnosis in the poor information condition, this paper proposed a data-driven fault diagnosis method that is based on SPIP and HMM. Perceptually Important Point technology is an adaptive time series compression method. The original signal can be transformed into PIP series that can depict the movement shape of the original signal clearly with little information loss. In order to improve the classification accuracy and computational efficiency, time series symbolization is implemented. All of the important points are encoded with a symbol according to their location in symbolic space. The space partition is based on mean value and standard deviation; the Genetic Algorithm is imported to search the optimal symbolic space partition scheme. The symbolic series obtained will serve as feature series of fault classification. Subsequently, HMM is used to recognize operating conditions, the advantage of HMM is that both symbol and transformation information can be utilized. The combination of SPIP and HMM can achieve good performance in poor information condition.

A fault simulation experiment is presented to validate the proposed method and the parameter optimization procedure is given. Four operating conditions and 600 samples for each condition are used to validate the performance. The results show that the fault classification accuracy reaches 99.625% when the length of PIP series is 10% of the length of the original signal and the symbolic space is divided into seven regions. The training process takes less than 1 min and the testing process takes less than 1.5 s. Subsequently, the method is compared with existing methods and the method can get no inferior performance to existing methods with samples only containing 250 points and signal duration is 0.025 s. The experiment shows that the proposed method can reach high classification accuracy with little information requirement. It has the ability to diagnose faults of the hydraulic pump in a practical operating process.

## Figures and Tables

**Table 1:** Perceptually Important Point algorithm.

**Algorithm 1: Perceptually Important Point**
Input: Original data series *X* = [*x*_1_, *x*_2_, *x*_3_, …, *x_n_*];
Target length of PIP series *m*;
Output: PIP series *P* = [*p*_1_, *p*_2_, *p*_3_, …, *p_m_*];
Begin;
Set *p*_1_ = *x*_1_; *p_m_*= *x_n_*;
Calculating vertical distance *d_i_* of remaining points *x_i_* in *X*;
Selecting *x_i_* with maximum vertical distance *d_i_* as *p*_3_;
Repeat until P all filled;
Arranging *P* according to the index of points in *X*;
Return PIP series *P*;
End;

**Table 2:** Symbolization algorithm.

**Algorithm 2: Symbolic algorithm**
Input: Original data series *X* = [*x*_1_, *x*_2_, *x*_3_, …, *x_n_*];
PIP series: *P* = [*p*_1_, *p*_2_, *p*_3_, …, *p_m_*];
Symbol set: *SY* = (*sy*_1_, *sy*_2_, …, *sy_k_*)
Output: Symbolic series *S* = [*s*_1_, *s*_2_, *s*_3_, …, *s_m_*];
Fractile series: *F* = [*f*_1_, *f*_2_, …, *f_k_*];
Begin;
Calculating mean value *μ* and standard deviation *σ* of original series;
Determining the number of region *k*;
Optimizing partition scheme with Genetic Algorithm;
Computing fractile series *F*;
Partition phase space with partition nodes;
Encoding important points according to their location in symbolic space.
For *i* = 1, *P_j_*∈ [*μ* − *f*_1_·*σ*, *μ* + *f*_1_·*σ*], *s_j_* = *sy*_1_;
For *i* > 1, *P_j_*∈ [*μ* + *f_i_*_−1_·*σ*, *μ* + *f_i_*·*σ*) ∪ *P_j_*∈ [*μ* − *f_i_*·*σ*, *μ* + *f_i_*_−1_·*σ*), *s_j_*= *sy_i_*;
Repeat until all the points in *P* are encoded;
Return symbolic series *S*;
End;

**Table 3:** Fault modes and sample partition.

	Fault Mode	Training Samples	Testing Samples	Signal Duration
Operating condition 1	normal	200	400	0.025s
Operating condition 2	Screw wearing 0.2 mm	200	400	0.025s
Operating condition 3	Screw wearing 0.4 mm	200	400	0.025s
Operating condition 4	Rolling bearing crack in inner race	200	400	0.025s

**Table 4:** Average Log likelihood outputted by models.

	Model 1	Model 2	Model 3	Model 4
Condition 1	−50.91	−60.35	−68.21	−73.34
Condition 2	−68.02	−57.29	−78.47	−82.83
Condition 3	−60.34	−61.97	−44.39	−57.21
Condition 4	−64.08	−73.32	−65.86	−45.98

**Table 5:** Confusion matrix of the method.

		Estimated Class			
		Condition 1	Condition 2	Condition 3	Condition 4
True class	Condition 1	398	2	0	0
	Condition 2	2	398	0	0
	Condition 3	0	0	399	1
	Condition 4	0	0	1	399

**Table 6:** Model’s performance with different number of symbol.

Number of Symbols	25 PIPs			50 PIPs		
	Training Time	Testing Time	Accuracy	Training Time	Testing Time	Accuracy
4	26.93	1.5529	97.875%	50.364	2.6857	98%
5	30.8365	2.2310	99.5%	55.6846	2.4327	99.875%
6	25.2357	1.2569	96.375%	60.5648	2.9680	98.625%
7	39.1613	1.4089	99.625%	55.6987	2.2786	99.25%
8	41.8503	1.3914	99.625%	68.9321	2.5137	99.625%
9	47.6847	1.7234	98.625%	82.3438	2.6525	99%

**Table 7:** Model’s property with different length of PIP series (seven symbols).

Length of PIP Series	Training Time	Testing Time	Reconstruction Error				Accuracy
			Condition 1	Condition 2	Condition 3	Condition 4	
10	16.8218	1.3258	0.9935	0.8056	1.2394	1.5299	91.125%
15	24.7076	1.5122	0.7119	0.6203	1.1112	1.2891	96.375%
20	37.2211	1.6450	0.6040	0.5168	1.0264	1.1272	98.625%
25	55.274	1.4089	0.5515	0.4491	0.9534	0.9699	99.625%
30	61.1820	2.0051	0.5175	0.4061	0.9039	0.8502	99.5%
35	75.2692	1.1486	0.4891	0.3736	0.8554	0.7608	99.375%
40	73.7563	1.2493	0.4633	0.3459	0.8101	0.6502	99.5%
45	83.1325	1.3547	0.4395	0.3223	0.7639	0.6116	99.5%
50	104.8866	1.6880	0.4176	0.3008	0.7198	0.5501	99%

**Table 8:** Performance of two similar feature extraction methods.

Classifier	Number of Testing Sample	Training Time	Testing Time	Signal Duration	Accuracy
SPIP + HMM (25 points)	300	39.2659	1.40	0.025 s	99.67%
SAX + HMM (25 points)	300	36.2714	2.43	0.025 s	97.33%
SAX + HMM (50 points)	300	62.1353	4.07	0.025 s	99%
ZC + HMM (25 intervals)	300	157.19	0.79	0.025 s	92.67%

**Table 9:** Performance of machine learning classifiers.

Classifier	Number of Testing Samples	Training Time	Testing Time	Signal Duration	Accuracy
SPIP + HMM	300	39.2659	1.40	0.025 s	99.67%
SVM	180	1.86	0.23	0.5 s	100%
RBFNN	180	-	0.2424	0.5 s	97.78%
BPNN	180	1.67	0.0922	0.5 s	88.88%
